# A Differential Genome-Wide Transcriptome Analysis: Impact of Cellular Copper on Complex Biological Processes like Aging and Development

**DOI:** 10.1371/journal.pone.0049292

**Published:** 2012-11-12

**Authors:** Jörg Servos, Andrea Hamann, Carolin Grimm, Heinz D. Osiewacz

**Affiliations:** Institute of Molecular Biosciences, Faculty for Biosciences & Cluster of Excellence ‘Macromolecular Complexes’, Johann Wolfgang Goethe University, Frankfurt, Germany; Auburn University, United States of America

## Abstract

The regulation of cellular copper homeostasis is crucial in biology. Impairments lead to severe dysfunctions and are known to affect aging and development. Previously, a loss-of-function mutation in the gene encoding the copper-sensing and copper-regulated transcription factor GRISEA of the filamentous fungus *Podospora anserina* was reported to lead to cellular copper depletion and a pleiotropic phenotype with hypopigmentation of the mycelium and the ascospores, affected fertility and increased lifespan by approximately 60% when compared to the wild type. This phenotype is linked to a switch from a copper-dependent standard to an alternative respiration leading to both a reduced generation of reactive oxygen species (ROS) and of adenosine triphosphate (ATP). We performed a genome-wide comparative transcriptome analysis of a wild-type strain and the copper-depleted grisea mutant. We unambiguously assigned 9,700 sequences of the transcriptome in both strains to the more than 10,600 predicted and annotated open reading frames of the *P. anserina* genome indicating 90% coverage of the transcriptome. 4,752 of the transcripts differed significantly in abundance with 1,156 transcripts differing at least 3-fold. Selected genes were investigated by qRT-PCR analyses. Apart from this general characterization we analyzed the data with special emphasis on molecular pathways related to the grisea mutation taking advantage of the available complete genomic sequence of *P. anserina*. This analysis verified but also corrected conclusions from earlier data obtained by single gene analysis, identified new candidates of factors as part of the cellular copper homeostasis system including target genes of transcription factor GRISEA, and provides a rich reference source of quantitative data for further in detail investigations. Overall, the present study demonstrates the importance of systems biology approaches also in cases were mutations in single genes are analyzed to explain the underlying mechanisms controlling complex biological processes like aging and development.

## Introduction

Copper is an essential micronutrient and a potential cytotoxic agent. These opposing features are due to the role of copper, a cofactor of different enzymes like cytochrome c oxidase (COX), superoxide dismutase 1 (SOD1), or laccase and tyrosinase, and the potential of Cu(II) to react with hydrogen peroxide to form the highly reactive and toxic hydroxyl radical. Therefore, a tight regulation of copper homeostasis is crucial to keep biological systems functional. Different molecular pathways including the controlled uptake of copper from the environment, the binding to chelators, and the efficient delivery of the metal to different cellular compartments and apoproteins are active. The transcriptional control of genes encoding copper-regulated transcription factors is one important molecular route.

In a series of previous investigations a role of copper in aging and development was demonstrated in the fungal aging model *Podospora anserina*. A mutant of this fungus in which the lifespan is increased by about 60% when compared to the wild type was found to lack the copper-regulated transcription factor GRISEA [1], [2]. At low cellular copper levels, GRISEA acts as a transcriptional activator of the *PaCtr3* gene encoding the high affinity copper transporter PaCTR3 [3]. At high cellular copper, the metal binds to GRISEA leading to a conformational change and a loss of transcription activity. In the grisea mutant this control is de-regulated [3]–[5]. The mutant is copper-depleted and copper-dependent activities like respiration, ROS scavenging and pigment biosynthesis are impaired. The lifespan increasing effect of the mutation appears to largely result from a switch from a copper-dependent to an iron-dependent alternative respiration which is characterized by a reduced generation of the superoxide anion at the respiratory chain and a stabilization of the mitochondrial genome [5]–[7]. However, other mutants respiring via the alternative pathway strongly differ in lifespan and examples of mutants are known which appear to be immortal (reviewed in: [8], [9]).

Investigations of the aging process in the wild-type strain of *P. anserina* revealed that the status of copper in the cytoplasm changes during aging [5]. Lateron this was confirmed for replicative aging of human fibroblasts in culture [10]. At least in *P. anserina* it appears very likely that copper becomes re-located from mitochondria, which previously were shown in yeast to contain a non-proteinacious copper pool used for metallation of apoproteins [11], [12], to the cytoplasm leading to age-related copper responses [3]. Collectively these data demonstrate an important role of copper in biological aging and development. However, the consequences of impairments in copper homeostasis are certainly not restricted to the components and molecular pathways identified so far. This conclusion can be drawn from the known effect of copper on the activity of specific transcription factors which directly control the transcription of different target genes. Moreover, indirect effects on gene expression can be expected to result from impairments in copper-dependent activities and the induction of compensatory responses. In order to obtain a more comprehensive insight into the genetic control of copper-affected pathways and processes, we performed a comparative genome-wide transcriptome analysis of wild-type strain ‘s’ and the long-lived copper depletion mutant grisea of *P. anserina* and investigated relevant molecular pathways on the basis of the available sequence of the complete genome.

## Results and Discussion

A single point mutation in the *Grisea* gene of *P. anserina* resulting in the loss of function of the copper-regulated GRISEA transcription factor was previously demonstrated to lead to cellular copper depletion and effects development and aging [13], [14]. Since copper is known to directly affect gene transcription via the interaction with transcription factors like GRISEA and indirectly to compensate impairments of copper-dependent functions like respiration or via affecting the turnover of transcripts and proteins, extensive differences in transcript profiles can be expected in the two investigated strains. However, up to now, in *P. anserina* only for a few genes effects of copper on the abundance of their transcripts have been shown. For many others such an impact is likely but remained to be demonstrated. For this purpose, we performed a differential genome-wide transcriptome analysis of the *P. anserina* wild type and the copper-depletion grisea mutant and linked the results to pathways deduced from available experimental data and the genomic sequence of this fungal model for aging and development. After germination of ascospores, the fungal isolates were first grown on solid medium for two days and afterwards in liquid medium again for two days to obtain the mycelia for RNA isolation. Thus, both strains were of identical chronological age, although the biological age, due to a lifespan extension of the grisea mutant, differs at that time-point.

### Overview of the Grisea Transcriptome Analysis

Overall a total of more than 7 million single tag molecules were sequenced for each of the two *P. anserina* strains, resulting in counts for 249,849 different tag sequences. By BLAST sequence comparison, 92,530 tag sequences could unambiguously be assigned to the sequence of one of the more than 10,600 predicted *P. anserina* coding sequences (CDS) annotated at the *P. anserina* genome database. This finally led to transcript data for 9,700 different *P. anserina* genes covering 90% of the transcriptome of this organism.

Comparison of the grisea and wild-type transcriptomes resulted in the identification of 4,752 of the 9,700 genes with a significant difference in abundance (confidence level p<0.01). Transcript levels of 1,156 of these genes were more than 3-fold different in the two strains. The abundance of 549 of these transcripts was lower in the grisea mutant and that of 607 transcripts was higher.

GRISEA has been demonstrated to be an ortholog of the yeast MAC1 transcription factor that has extensively been investigated [15]–[17] and shown to be a copper-sensing transcription factor that, at low copper levels, activates the transcription of genes coding for two copper transporters. Among the genes for which transcripts were identified to differ in abundance under copper-repleted or depleted conditions there are GRISEA target genes but also those controlled by yet unknown transcription factors which are regulated by copper or other signals like ROS. Thus, the results of the presented transcriptome analysis alone do not allow the definitive identification of genes regulated by the GRISEA transcription factor. In addition, the observed differences in transcript abundance may also result from differential RNA turn-over under copper-repleted vs. depleted conditions.

Analyzing the present transcription profiling experiment for GO terms which are assigned to differentially expressed *P. anserina* genes at a higher level than expected by chance, revealed the most significant enrichments of GO terms connected to mitochondria. As shown in Table S1, the GO terms ‘*mitochondrion*’, ‘*mitochondrial part’*, ‘*mitochondrion organization’*, ‘*mitochondrial translation’*, ‘*mitochondrial matrix’* and ‘*mitochondrial envelope’* are the six most significantly enriched GO-terms (lowest GO enrichment p values) found among the differentially expressed *P. anserina* genes comparing grisea mutant strains and the wild type. This finding is consistent with previously described differences of the respiratory chain of the grisea mutant strain which respires via the alternative respiratory pathway leading to a bioenergetics impairments. The reduction in ATP generation may explain a general down-regulation of gene expression in the grisea.

### Copper Metabolism

The grisea mutant was previously described as a mutant affected in copper-uptake. The mutant phenotype can be rescued to wild-type characteristics by increasing copper concentrations in the growth medium [14]. The phenotype results from a loss-of-function mutation in the *Grisea* gene which encodes the copper-regulated transcription factor GRISEA that is an ortholog of the MAC1 yeast transcription factor and is involved in the control of copper-uptake [1], [2]. Two putative copper transporters, PaCTR2 and PaCTR3, were experimentally demonstrated to rescue a copper-uptake mutant of *S. cerevisiae* in which two high affinity transporters ScCTR1 and ScCTR3 are absent. Rescue from copper depletion was more efficient by PaCTR3 than PaCTR2 and led to the conclusion that the two proteins encode a high and a low affinity copper transporter of *P. anserina*, respectively. *PaCtr3* expression was found to depend on the presence of the active GRISEA transcription factor. In the grisea mutant and in the wild type grown on copper supplemented medium *PaCtr3* transcripts levels are very low to undetectable [3]. A third open reading frame (*Pa_1_16400*) is encoding a putative protein with high sequence similarity to the *S. cerevisiae* CTR3 protein (blast e = 2E−21). It is annotated in the *P. anserina* gene database [18] as a gene predicted to code for a ‘putative copper transport protein’. No experimental data about the expression and regulation of *PaCtr2* and of the putative *Pa_1_16400* gene were available until now.

Here we report that transcript levels of both genes are higher in the wild type than in the copper- depleted grisea mutant (Table S2). Since the *Pa_1_16400* gene is differentially expressed in a copper dependent manner, the corresponding protein bears significant homology to copper transporters, and since PaCTR2 and PaCTR3 have already been allocated to the two proteins encoded by two genes selected in yeast complementation assays, we now assign the *Pa_1_16400* encoded protein as PaCTR1.

**Figure 1:**
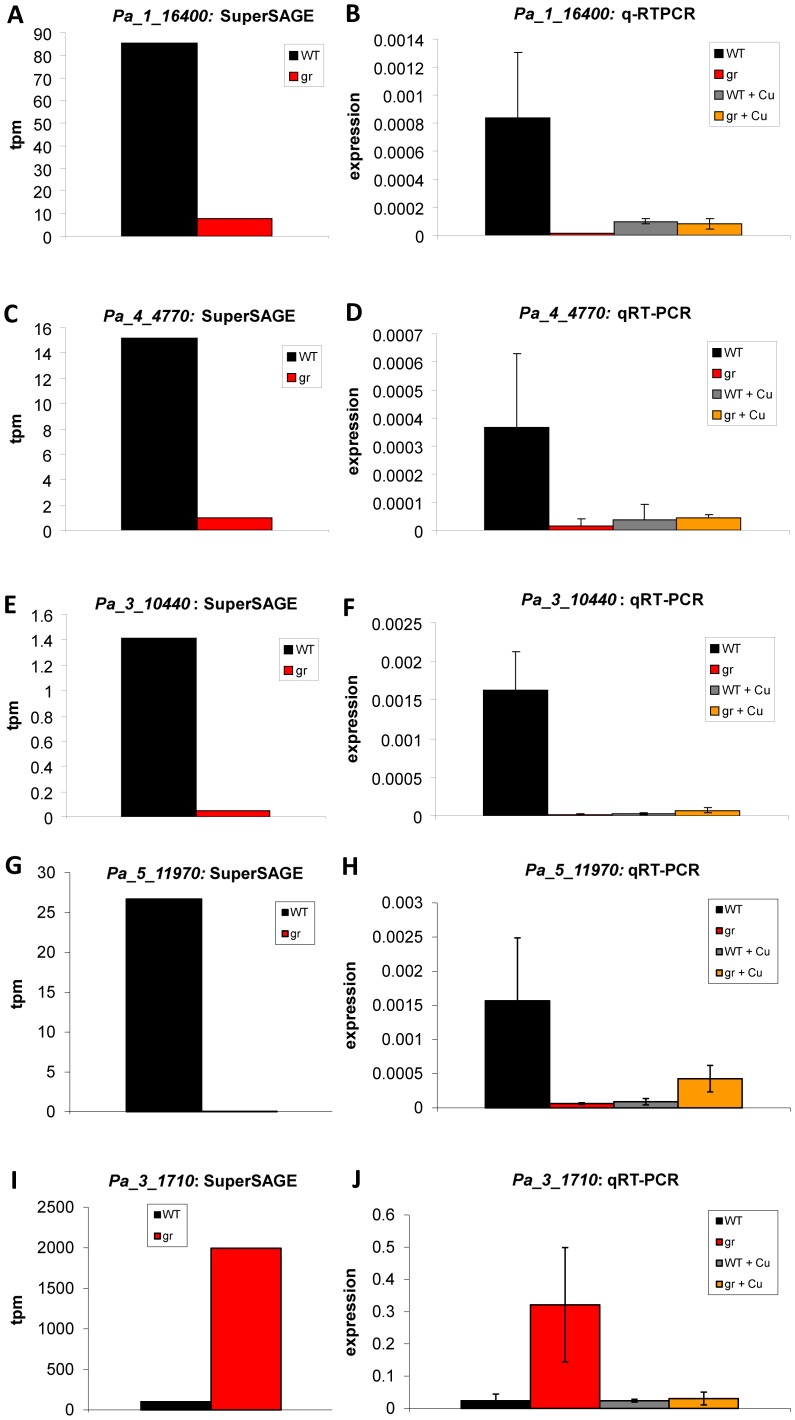
Transcript analysis of *Pa_1_16400* (*PaCtr1*: A, B), *Pa_4_4770* (*PaCtr2*: C, D), and *Pa_3_10440* (*PaCtr3*: E, F), *Pa_5_11970* (G, H), *Pa_3_1710* (I, J). Results from the SuperSAGE analysis (**A, C, E, G, I**) are shown as ‘tags per million’ (tpm) for wild type (WT) and grisea (gr). The results of the qRT-PCR (**B, D, F, H, J**) are relative expression levels of the three copper transporter genes of the wild type (WT, n = 5 in **B**, **D**, **F**, **J**; n = 6 in **H**) and the grisea mutant (gr, n = 6) grown in standard medium, and wild type (WT+Cu, n = 3) and grisea (gr+Cu, n = 3) grown on copper-supplemented medium, normalized to the expression level of the gene coding for mitochondrial PORIN The error bars represent the standard deviation.

As an important control of the SuperSAGE transcriptome analysis of this study, the results from previous Northern Blot analyses of *PaCtr3* [3] were confirmed. While *PaCtr3* transcripts were counted as 1.41 transcripts per million (tpm) in the wild type, no transcript (<0.05) was detected in the grisea mutant strain (Table S2). To further validate the results obtained in the SuperSAGE analysis, and to demonstrate their biological relevance, quantitative real time PCR (qRT-PCR) experiments were performed. The same samples were used as those in the SuperSAGE analyses but in addition newly isolated samples from wild-type and grisea cultures (biological replicates) were included in this study. In Fig. 1, the results of the SuperSAGE analysis (Fig. 1A, C, E) and the qRT-PCR (Fig. 1B, D, F) are compared. Obviously, the two different techniques revealed consistent results both with highly abundant (Fig. 1 I and J) and rare transcripts (Fig. 1 A and B, C and D).

**Figure 2:**
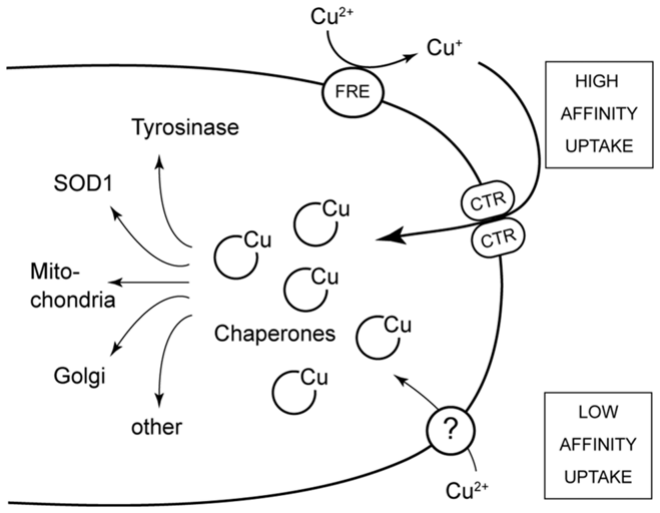
High and low affinity copper uptake of copper and delivery to different enzymes and compartments. At cellular copper levels, copper-uptake is known from *S. cerevisiae* and *P. anserina* to involve high affinity uptake mechanism. After reduction of copper mediated by a membrane located oxidoreductase (FRE) the import is performed by high affinity copper transporters (CTR). Expression of CTR-transporter genes is regulated transcriptionally by copper-dependent transcription factors. Intracellularly the copper is stored and distributed via copper binding chaperones and partially becomes transported into mitochondrion where it is stored and distributed to copper-containing proteins (i.e. cytochrome oxidase). Copper is also taken up by cells via a low copper-uptake system.

Since GRISEA is a functional homolog of MAC1 and since this yeast transcription factor is regulating the two genes coding for the two high affinity copper transporters ScCTR1 and ScCTR3, we assumed that both *P. anserina* genes, *PaCtr1* and *PaCtr3*, are controlled by GRISEA. In this case, the expression of both genes should remain repressed in the grisea mutant even in the presence of excess copper in the growth medium. In contrast, if both genes are down-regulated in the mutant due to copper-deficiency, treatment with copper should restore the transcript abundance to wild type levels. As can be seen from Figs. 1B and F, the relative expression level of both genes remains low in grisea strains treated with copper, clearly demonstrating that both genes are GRISEA targets. Because of sequence conservation and since PaCTR3 was previously shown to efficiently rescue the yeast CTR1/3 deficiency mutant, we further propose that both genes encode potential high affinity copper transporters (Fig. 2). Failure to select *PaCtr1* in previous complementation experiments is probably due to the lower expression of this gene (Fig. 1B and F) in *P. anserina* resulting in an under-representation of this gene in the *P. anserina* cDNA library used for the complementation.

Interestingly, in the genome-wide transcriptome analysis the abundance of *PaCtr2* transcripts, encoded by the gene which was first suggested to code for a homolog of ScCTR2, representing a low affinity copper transporter and later found to be localized in vacuolar membranes, are strongly reduced in the grisea mutant. Also for this *P. anserina* gene excess copper is not able to restore the transcript levels in the grisea mutant to those of the wild type (Fig. 1D). Thus, in contrast to *ScCtr2* in yeast that is regulated independent of MAC1, it is very likely, that *PaCtr2* encodes another copper transporter that is regulated by transcription factor GRISEA.

A good candidate for a gene coding for a copper transporter acting as an ortholog of the yeast vacuolar copper transporter ScCTR2 the expression of which is not activated by transcription factor GRISEA is the putative product encoded by *Pa_1_4220*. This protein shows the highest sequence similarity to the yeast ScCTR2 (blast e = 4E−17) and is regulated independent of GRISEA since transcript levels in the grisea mutant are about 1.49-fold higher than in the wild type (Table S2).

### Iron Metabolism

**Figure 3:**
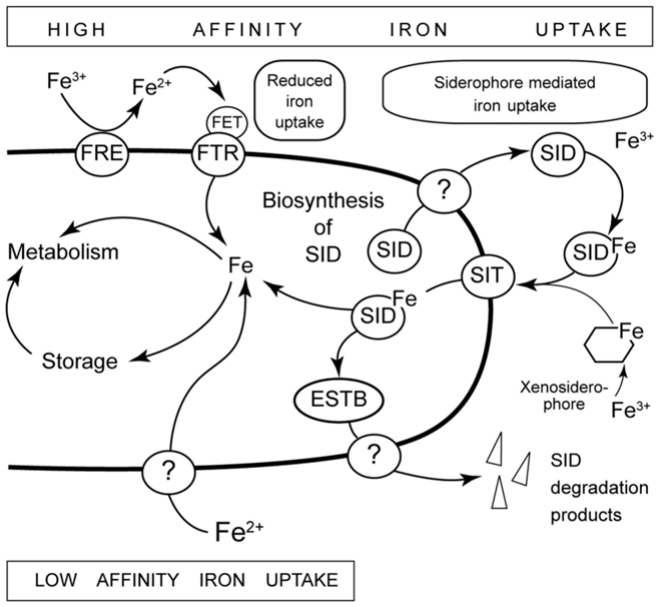
High and low affinity uptake of iron in filamentous fungi. Under iron limiting conditions two high affinity iron import mechanisms are involved in uptake of iron from the environment. Via the reductive iron assimilation (RIA) system, ferric iron first becomes reduced to ferrous iron by a reductase (FRE) and then oxidized by a multicopper oxidase (FET) and imported by a plasma membrane transporter (FTR). Many fungi also produce and secrete siderophores (SID) and, after binding of iron (SID Fe) are reimported into the cell. Bound iron is subsequently made accessible to the cell by activity of a specific esterase (ESTB). In *S. cerevisiae* is only able to take up siderophores (Xenosiderophores) that available in the environment.

In fungi different iron-uptake systems have been described (reviewed in: [19]). Under iron-limiting conditions two high affinity iron uptake systems are essential to supply cells with sufficient amounts of the metal (Fig. 3). One system, termed reductive iron assimilation (RIA), is dependent on the reduction of ferric iron (Fe^3+^) to ferrous iron (Fe^2+^) and the uptake via a plasma membrane transporter. In yeast, membrane bound ScFRE1, encoded by the MAC1 target gene *ScFre1*, is the active reductase. The high affinity iron transporter ScFTR1 is associated with the multicopper oxidase ScFET3 which prior to transport across the membrane oxidizes ferrous iron to ferric iron. A second high affinity uptake pathway works via siderophores (SID). These low molecular mass organic molecules with high affinity for iron are synthesized and secreted by the fungus, chelate iron and are subsequently taken up by transporters (SIT) in the plasma membrane. Although *S. cerevisiae* is able to take up siderophores present in the growth medium (xenosiderophores), it does not synthesize and secrete these compounds. Thus, under xenosiderophore-free laboratory conditions high affinity copper-uptake in yeast is restricted to RIA and thus is dependent on the availability of enough copper inside the cell to load the multicopper oxidase ScFET3. Consequently, in yeast, copper deficiency leads to iron-depletion as well. In filamentous fungi this is not the case because they appear to encode all components of both high affinity uptake systems. Both systems do not need to be active at the same time but activity may be controlled by environmental necessities (for review see: [19]). The gene repertoire and the data from our transcriptome analysis support the activity of two high iron affinity uptake systems in *P. anserina*.

### High Affinity Iron Uptake in *P. anserina* by Reductive Iron Assimilation (RIA)

The evaluation of the *P. anserina* genome database revealed all potential components required for a RIA pathway as they are known from yeast. The results of the analysis are compiled in Table S3. Six putative genes are annotated to code for ‘putative ferric reductase transmembrane components’. Transcript levels of one of these genes, *Pa_1_19550*, are higher in the grisea mutant than in the wild type and therefore the gene appears not to be activated by GRISEA. One of the encoded proteins (Pa_5_11970) shares a very high homology to both of the two yeast proteins FRE1, and FRE2 (blast e-values 9.0E-32 and 8.0E-30, respectively). Since sequence homology is higher with ScFRE1, we annotate the corresponding gene as *PaFre1*. Similar to *PaCtr3*, no transcripts are found in the grisea mutant. The same result is observed in the qRT-PCR analysis of this gene (Fig. 1 G, H). In yeast, *ScFre1* is controlled by the GRISEA homolog MAC1. However, since addition of copper to mutant grisea leads to an induction of the transcript abundance (Fig. 1 H), this gene seems only to be partly regulated by GRISEA. Other regulator proteins may also affect the transcription in the absence of GRISEA.

There are four additional potential metalloproteases encoding genes found in the *P. anserina* genome. No transcripts of *Pa_5_10230* were identified in the wild type and the grisea mutant suggesting that the corresponding gene, at least under the conditions the corresponding strains were cultured, is not expressed. In contrast, transcript levels of *Pa_7_5660*, *Pa_1_19630*, and *Pa_1_16410* were found to be lower in the grisea mutant strain than in the wild type with ratios of 0.68, 0.44 and 0.21, respectively. Because these differences are not as pronounced as for the genes that most likely are targets of GRISEA, we do not assign these genes as direct targets of GRISEA.

In the *P. anserina* genome one ORF, *Pa_6_4210* has been suggested to code for a putative ‘plasma membrane iron permease’ with high homology to ScFTR1 from yeast. The gene is transcribed and measured transcript levels are higher in the wild type than in the grisea mutant. However, due to the low number of transcripts counted for this gene in the SuperSAGE analysis, this difference was mathematically not significant.

Finally, several multicopper oxidases have been annotated in the *P. anserina* database. One of them shares sequence homology with ScFET3, the oxidase involved in iron-uptake in yeast (Pa_6_4220 with blast e-values of 1E−171). Remarkably, no or very few transcripts were identified for this putative gene in the wild type and in the grisea mutant, respectively. Among the next three genes encoding proteins with homology to ScFET3, transcripts of one gene (*Pa_2_530*) are found to be much lower in the grisea mutant than in the wild type. The corresponding gene is annotated to code for a putative iron transport multicopper oxidase. Of the other two genes, one is annotated to code for a putative multicopper oxidase (Pa_6_2550) and one for a ‘laccase-2 precursor’ (Pa_5_1200) [20] and as such potentially linked to pigment biosynthesis. Considering the blast e-values from 4E-45 to 1E-26, there are eight additional genes present in the *P. anserina* genome annotated as putative laccases sharing sequence homology with ScFET3 (data not shown). Although there is clear coding potential for multicopper oxidase involved in high affinity iron uptake via RIA we currently do not know which one is the functional protein in *P. anserina* and we also do not know under which conditions the corresponding gene is expressed.

#### High affinity siderophore mediated iron uptake

Like most other fungal species, *P. anserina* appears not to code for an ortholog of the yeast iron sensitive ScAFT1 regulator protein, which is the primary iron regulated transcriptional activator of genes encoding iron-uptake activities in *S. cerevisiae* [21], [22]. Moreover, *P. anserina* does not appear to encode an ortholog of the *A. fumigatus* SreA protein, which is known to be involved in regulation of siderophore mediated iron transport [23]. In *A. nidulans*, transcription of genes encoding the siderophore transporters MirA, MirB and MirC and of the orthologs of the *A. fumigatus* SidA and SidC proteins is repressed by iron, mediated by the negative-acting transcription factor SreA. Also in *A. fumigatus*, the orthologous protein is known to release its repression on genes involved in siderophore production and iron uptake under iron-depleted conditions [24]–[27]. Accordingly, the predicted *P. anserina* ortholog of the *A. fumigatus* SREA protein, Pa_6_1720 is annotated as a ‘putative siderophore regulation protein’ (Table S4). Transcript molecules of the respective reading frame have been detected in the present analysis in both the wild-type and the grisea mutant strain with no obvious difference of expression levels. Transcription of the respective reading frame has been detected in the present analysis and expression of this gene is found to be reduced in grisea mutant strains to half of the wild types level. In *A. fumigatus*, expression of the four genes *AfSidC*, *AfSidF*, *AfSidD* and *AfSidG* of the siderophore pathway was shown to be activated by iron depletion to facilitate iron siderophore production. The extracellular siderophore fusarinine C (FC) is synthesized by a biosynthetic pathways involving the consecutive action of the proteins encoded by the genes *AfsidA*, *AfsidF*, and *AfsidD*. An additional protein encoded by the gene *AfsidG* is involved to produce the siderophore triacetylfusarinine C (TAFC). The produced siderophore gets excreted by specific siderophore transport proteins (SIT) [28]. Subsequent to the extracellular binding of iron and uptake of the siderophore-iron complex, the specific TAFC esterase EstB, encoded by the gene AfEstB, releases iron by intracellular degradation of the siderophore [29].

In Table S4, proteins of siderophore mediated iron transport of *A. fumigatus* and putative ortholog proteins of *P. anserina* are juxtaposed. For most of the known proteins of the *Aspergillus* siderophore synthesis pathway we identified at least one putative homolog in *P. anserina* by genomic data mining. Especially the two putative non-ribosomal peptide synthetases SidC and SidD of *A. fumigatus*, which are thought to be involved in biosynthesis of the siderophores ferricrocin (FC) and triacetylfusarinine C (TAFC), share very high sequence similarity to more than one predicted *P. anserina* protein, each (Pa_4_4440, Pa_4_4640, and Pa_2_7870 for AfSidC and Pa_3_11200, Pa_4_4640, and Pa_5_1070 for AfSidD). This has also been discussed before to be the case for the SIDA ornithine-N-monooxygenase, which seems to be present in all Pezizomycotina in up to three paralogs [19]. The enzyme is missing in species not able to produce siderophores, like *S. cerevisiae*, *C. albicans*, and *C. neoformans* [30], [31]. No obvious ortholog of the *A. fumigatus* SidG acetyl transferase could be identified in the *P. anserina* genome sequence. In fact this protein, which converts FC into TAFC, is only found in some fungal species. In *A. fumigatus*, SidG-deficient strains lack TAFC, but instead produce FC as the major siderophore. FC seems to fully compensate for the lack of TAFC, since such mutants are phenotypically indistinguishable from the wild type [19].

Taken together, in contrast to *S. cerevisiae*, *P. anserina* contains the complete machinery for iron-uptake via siderophores and encodes the components necessary to synthesize siderophores for secretion. Transcript levels of some siderophore-related genes are higher in the grisea mutant than in the wild type (Table S4). This increase may be a compensation of impairments of iron-uptake via RIA as a result of copper limitation.

### Energy Metabolism

#### Catabolic processes

Among the transcripts which are increased above 3-fold, several transcript encoding enzymes involved in catabolic processes were identified (Table S5). Among these, transcripts of a putative glucokinase, an enzyme of glycolysis, is increased 3.13-fold in abundance in the grisea mutant strain. The genes encoding the putative aconitase of *P. anserina* (Pa_2_12390) and the putative fumarate hydratase (Pa_1_19320), both enzymes of the krebs cycle, were found to be increased 3.09-fold and 4.33-fold, respectively. In contrast, in grisea the transcripts of the gene encoding the putative 2-methylcitrate synthase of *P. anserina* (Pa_6_10000) are decreased to 8% of that in the wild type. This differential transcript abundance is similar to the two most likely targets of GRISEA, *PaCtr3* and *PaFtr1*, and the corresponding gene therefore represents another potential target of the GRISEA transcription factor.

#### Respiration

**Figure 4:**
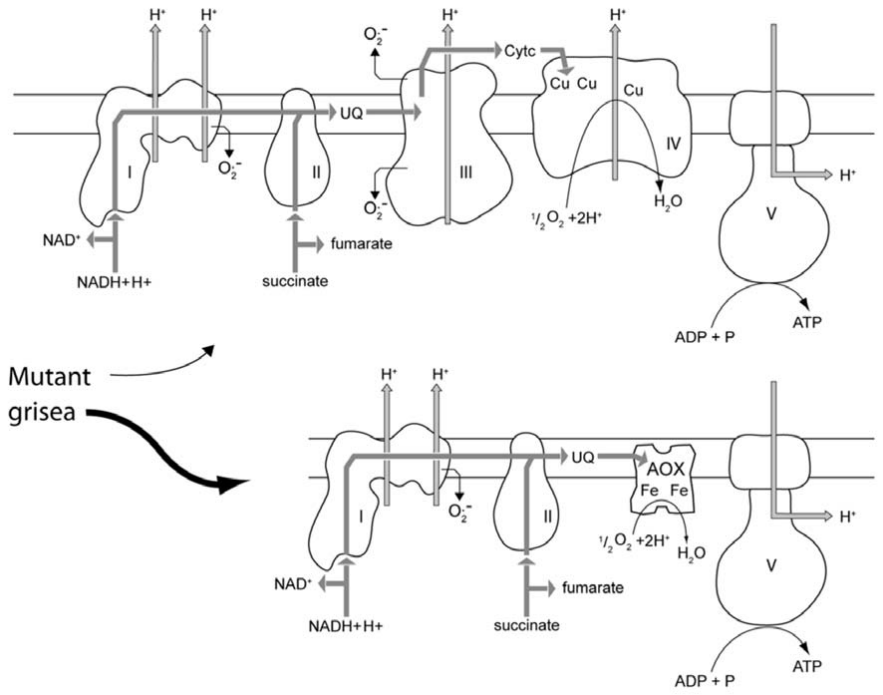
Copper-dependent standard and alternative respiration in *P. anserina*. While respiration of the wild type predominantly proceeds via the standard copper-dependent pathway, respiration in the grisea mutants does mainly follow the alternative route utilizing the di-iron containing alternative oxidase.

The previous demonstration of an alternative respiration in the copper-uptake mutant grisea was strong evidence for a copper-independent high affinity iron-uptake system in *P. anserina* [3], [5]. This type of respiration utilizes the terminal oxidase PaAOX1 (Pa_3_1710) and di-iron instead of copper for delivery of electrons from the respiratory chain to oxygen enabling *P. anserina* to respire under copper-depleted conditions (Fig. 4). The electron transfer via AOX was found to result in reduced levels of reactive oxygen species (ROS) and is considered to play a key role in the enhanced lifespan phenotype of grisea mutant strains [32]. Northern Blot experiments revealed that cellular copper depletion as a result of growth of the wild type on medium containing a copper chelator or as a defect in copper-uptake leads to an induction of *PaAox1* (Pa_3_1710) and a strong increase in transcript abundance [5]. Again, these data were verified in the present study in which *PaAox1* transcript abundance was found to be 19.81-fold higher in the grisea mutant (Table S6). The subsequent qRT-PCR analysis of this gene not only confirmed these data but also demonstrated that growth in copper-supplemented medium nearly completely abolishes *PaAox1* expression in the grisea mutant (Fig. 1 I, J). Thus, the expression of this gene is repressed by copper.

Since in the alternative pathway electrons are directly transferred from ubichinol to the alternative oxidase and complexes III and IV are by-passed (Fig. 4), it appeared reasonable to expect that transcription of components of complexes III and IV is down-regulated. However, the data from our study did not provide clear evidence for such a scenario (Table S6). While transcripts of the mitochondrial encoded genes *PaCox2* and *PaCox3* were lower in abundance in the grisea mutant, other transcripts of this complex, like those of *PaCox1* or some genes encoding proteins of complexes III and IV, were either not significantly changed or increased in abundance. It appears that there is only a moderate correlation between the kind of respiration and the abundance of transcripts of individual protein components.

Previously, it has been noticed that, although the grisea mutant respires via an alternative respiration that leads to the generation of less ATP than the standard copper-dependent respiration of the wild type, the growth rate was observed to be almost identical to that of the wild-type strain. This was correlated with an increased oxygen consumption and greater mitochondrial mass suggesting that during the years of sub-cultivation in the laboratory a compensatory effect occurred [33]. The transcriptome data do not demonstrate a clear correlation of transcript levels, the mode of respiration and of compensatory effects due to an energetically less efficient alternative respiration. These data suggest that, although it may be important to increase or decrease certain transcripts under certain conditions subsequent post-transcriptional processes (e.g., translational efficiency, protein degradation during complex assembly) may play a key role in triggering the efficiency of complex molecular pathways.

### ROS Detoxification

Respiration is a main generator of reactive oxygen species (ROS) and as such the kernel of the ‘mitochondrial free radical theory of aging’ which states that aging results from the accumulation of molecular damage induced by ROS generated by mitochondria [34]. Previously it was demonstrated that the lifespan extension of the grisea mutant is linked to a switch from the standard copper-dependent respiration to an alternative, iron-dependent respiration and a strongly reduced generation of the superoxide anion [5], [7]. However, compared to the wild type and a number of other long-lived mutants respiring via an alternative respiration the lifespan of this mutant is only moderately increased (for review see: [35], [36]). One of the reasons for the limited lifespan increase may be impairments in copper supply in mutant grisea and its impact on ROS detoxification. This view is supported by a specific mutant in which *PaCox17*, coding for a copper chaperone that delivers the metal to cytochrome oxidase, was deleted [37]. Like in the grisea mutant, respiration is switched to the alternative pathway but, unlike in grisea, cellular copper levels are not affected in the *PaCox17* deletion mutant and Cu/Zn SOD activity is unaffected in the *PaCox17* deletion strain. The lifespan of this mutant is much longer than that of the grisea mutant.

**Figure 5:**
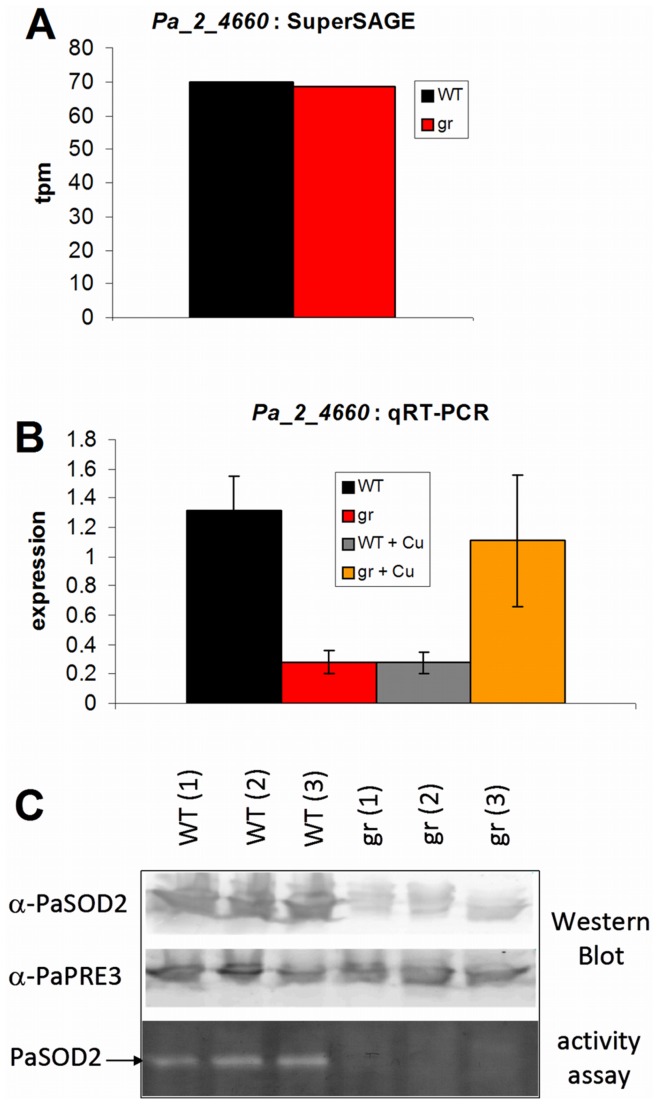
Transcript (A, B) and protein analysis (C) of *Pa_2_4660* (*PaSod2*). The results of the SuperSAGE analysis (**A**) are shown as tags per million (tpm) for wild type (WT) and grisea (gr). The results of the qRT-PCR (**B**) are relative expression levels of the gene of the wild type (WT, n = 5) and of grisea (gr, n = 6) grown in standard medium, and wild type (WT+Cu, n = 3) and grisea (gr+Cu, n = 3) grown on copper supplemented medium, normalized to the expression level of gene coding for mitochondrial PORIN. The error bars represent the standard deviation. For Western Blot (α-PaSOD2) and *in-gel* SOD activity assay (**C**) 100 µg of total protein extracts of three independent wild type (WT) and grisea (gr) isolates were used. As loading control the α-PaPRE3 antibody was used.

The data from our transcriptome analysis revealed that transcript abundance of genes coding for different components of the ROS scavenging machinery does not dramatically differ between wild type and the grisea mutant. In the grisea mutant, the strongest increase of transcripts was found for a gene annotated to code for a ‘putative mitochondrial SSU ribosomal protein S26 precursor’ (Table S7). This putative protein (Pa_1_10620) shares strong sequence similarities to several superoxide dismutases of other organisms like, e.g. the manganese and iron superoxide dismutase of *Neurospora tetrasperma* (UniProt G4UBU2, blast e = 1E−58). The protein is predicted by the *InterProScan* tool [38] to possess metal ion binding and superoxide dismutase activity. Thus, this protein appears to represent a yet unknown SOD of *P. anserina*. Transcript levels of two of the three SODs previously described for *P. anserina* were only moderately increased with a slight increase of *PaSod1* and *PaSod3* transcripts in the grisea mutant while those of *PaSod2* are unchanged. The latter finding was unexpected because, on the basis of previous RT-PCR experiments with degenerated primers deduced from sequences of a MnSOD from related fungi, *PaSod2* was concluded to be a GRISEA target and not expressed in the grisea mutant [5]. The current analysis revealed contradictory results, since now it was found that the transcript level is not significantly changed in the mutant (Fig. 5A). The qRT-PCR analysis demonstrates lower transcript abundance of the gene in the grisea mutant (Fig. 5B), but the transcription is not completely abolished. To further validate these new data, the amount as well as the activity of PaSOD2 was determined (Fig. 5C). As described earlier [5], the mutant lacks PaSOD2 *in-gel* activity. The lack of detectable activity seems to be due to the much lower amount of PaSOD2 protein in the mutant: although nearly equal amounts of total protein extract were used (Fig. 5C, loading control with anti-PaPRE3 antibody), much less PaSOD2 protein is detectable in the mutant. However, as shown in Fig. 5B, since copper addition to the mutant grisea restores the transcription of *PaSod2*, the gene is clearly not regulated by GRISEA, but by copper deficiency.

Moreover, we demonstrated in another recent study that PaSOD2 is not the mitochondrial SOD but most like to be a secreted isoform and that the mitochondrial isoform is encoded by *PaSod3* [39]. In earlier Northern analysis experiments we concluded a constitutive expression of *PaSod1*, the cytoplasmic and mitochondrial intermembrane space SOD, during aging in both the wild type and the grisea mutant. This was in contrast to the situation in yeast, where *ScSod1* is transcriptionally regulated under the control of a copper-regulated transcription repressor ScACE1. A homolog of ScACE1 has not been unambiguously identified.

**Figure 6:**
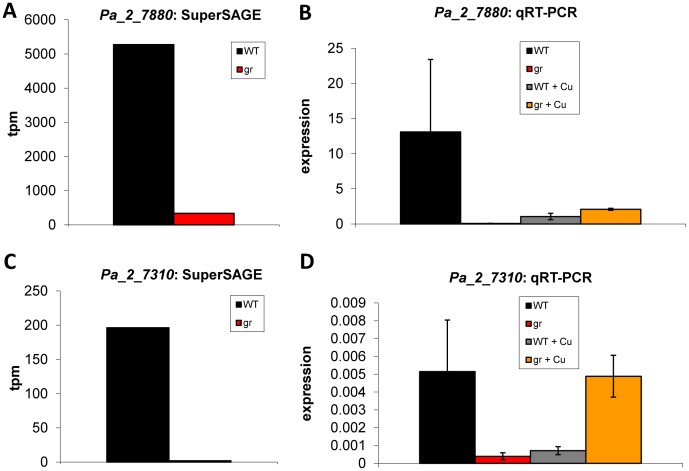
Transcript analysis of *Pa_2_7880* (*PaMth1*: A, B) and *Pa_2_7310* (C, D). The results of the SuperSAGE analysis (**A, C**) are shown as tags per million (tpm) for wild type (WT) and grisea (gr). The results of the qRT-PCR (**B, D**) are relative expression levels of the gene of the wild type (WT, n = 6) and of grisea (gr, n = 6) grown in standard medium, and wild type (WT+Cu, n = 3) and grisea (gr+Cu, n = 3) grown on copper supplemented medium, normalized to the expression level of the gene coding for mitochondrial PORIN. The error bars represent the standard deviation.

Transcription of the gene encoding the putative O-methyltransferase PaMTH1 (Pa_2_7880) is in the present SuperSAGE analysis found to be significantly reduced in the grisea mutant strain when compared to the wild type (Table S7, Fig. 6A). The gene was previously reported to be differentially expressed during aging [40]. The encoded protein is thought to be active in the prevention of ROS dependent damage of polyphenols containing vicinal hydroxyl groups. PaMTH1 accumulates in the mitochondrial fraction of cell lysates from senescent cultures and over-expression of *PaMth1* leads to an extended lifespan without impairing viability [41], [42]. The qRT-PCR analysis confirmed the down-regulation of *PaMth1* in the mutant (Fig. 6B), but does also demonstrate that addition of copper is not able to restore transcription (Fig. 6B, gr+Cu). This strongly supports a GRISEA-dependent transcriptional regulation of this gene.

The transcriptome data related to other ROS scavenging enzymes including catalases, cytochrome c peroxidase, or peroxiredoxin, show only a moderate increase in transcript levels in the grisea mutant that might contribute to lifespan extension. Only for peroxisomal peroxiredoxin an increase in transcript levels above the threshold of three was observed in this study.

One of the striking phenotypic changes of the grisea mutant is the reduced pigmentation of the mycelium and the ascospores that can be reverted by growth of strains on copper-supplemented medium. In fungi, the formation of melanins via the DHN pathway (named for one of the pathway intermediates, 1,8-dihydroxynaphthalene) and the DOPA pathway (named for one of the precursors, L-3,4-dihydroxyphenylalanine) is depending on copper as a cofactor of tyrosinase and laccases which catalyze individual steps of the biosynthesis pathways. Both DOPA- and DHN-melanins are able to quench free radicals and thus have ROS protection capacity. They also appear to be an important factor in virulence of pathogenic fungi [43].

Except for those of the last two reactions, all genes encoding components of the *P. anserina* DHN-melanin synthesis pathways have been reported in an earlier study [44]. Mutation of the melanin biosynthesis polyketide synthase PaPKS1 was found to result in unpigmented mycelia and perithecia at all stages of its life cycle [44]. The phenotype of this strain can be restored to wild-type pigmentation by addition of scytalone to the growth medium, which is an intermediate of the DHN-melanin synthesis pathway. The data from the current transcriptome study support this pathway as being active in pigment formation. In the hypopigmentation grisea mutant, the level of the PaPKS1 transcript (*Pa_2_510*) is decreased to 38% of the level in the wild type. In addition, transcripts of other previously identified potential genes involved in the DHN-melanin are significantly decreased in abundance in the mutant strain (Table S8). However, in *P. anserina* it is yet not known which specific enzymes are catalyzing the last two steps of the DHN-melanin pathway [44]. In *A. fumigatus*, these reactions require a vermelone dehydratase and a copper-containing oxidase (laccase) encoded by the *A. fumigatus* genes *Abr1* and *Abr2*, respectively [45]. The two *P. anserina* proteins showing the strongest sequence similarities to the product of the *A. fumigatus Abr1* gene are Pa_6_4220 (blast e = 1E−97), which is annotated in the *P. anserina* genome database as a putative ortholog of the yeast FET3 multicopper oxidase involved in iron transport, and Pa_2_530 (blast e = 1E−90), which is annotated as another putative ortholog of the yeast FET3 protein. The gene encoding the latter protein shows a decreased transcription in the grisea mutant strain of 8% when compared to the wild types transcription level (Table S3). In the genome database of *P. anserina* there are nine genes annotated to encode putative laccase precursors. Transcripts from six of them have been identified in the present transcriptome analysis (Table S8). Interestingly, transcripts of three of the predicted laccase precursor genes, *Pa_1_15470*, *Pa_5_4140*, *Pa_6_7880*, are highly abundant in the wild type and strongly reduced in the grisea mutant. Since laccases are multicopper oxidases, their impairment resulting from copper depletion in the grisea mutant makes these three proteins good candidates for components of the DHN melanin pathway being responsible for the observed hypopigmentation of the grisea mutant and the reported impairments in laccase activity reported for the grisea mutant [13], [46]. Consistently, wild-type strains of *P. anserina* develop a grisea-like phenotype when grown on copper-depleted media [14]. Variation of copper levels in the growth medium has been found to lead to differences in pigmentation for many fungal species and was shown to correlate with changes in laccase activity [47]. In *C. neoformans*, copper stimulates laccase gene expression [48], [49].

Overall our analysis supports the role of the DHN-melanin synthesis pathway in *P. anserina* and shows that the abundance of the transcripts of several components, but not of all (e.g., *Pa_5_1200*), of this pathway is copper-dependent. It is interesting, that transcripts abundance of some genes coding for laccases, a group of multicopper oxidases, differ under copper-replete and deplete conditions. The regulation of these enzymes may occur in a copper-dependent way at the transcriptional level while other laccases may be regulated at the protein level. It will be interesting to elucidate the details in the regulation mechanisms and, since laccases are not only involved in pigment synthesis but also in other functions like polymerization and depolymerization of lignin, to link them to specific enzymes and functions.

### Protein Quality Control

In *P. anserina* different proteases have been demonstrated to keep mitochondria functional and play a protective role [50], [51]. While not demonstrated in detail for *P. anserina*, this appears to hold true also for heat shock proteins. The components of the protein quality control system affect the pace by which aging processes proceed and thereby have a strong impact on lifespan [50], [52], [53]. The mitochondrial *P. anserina* LON protease PaLON1 (Pa_3_4170) and the chaperone PaHSP60 (Pa_6_5750) were recently shown to differ in abundance in the wild type and grisea mutant with increased proteins present in the grisea strain [33]. In the present study we found that the increase in protein abundance is correlated with a significant increase in transcript levels of the two genes encoding these proteins (Table S9). In addition, the amount of transcripts for the *P. anserina* chaperone SSC1, the mitochondrial HSP70 homolog (Pa_6_2570), and for m-AAA (Pa_2_5010), a protease of the inner mitochondrial membrane, was found in the present analysis to be strongly increased in the grisea mutant. Both proteins are thought to be involved in mitochondrial quality control in *P. anserina*. A moderate increase of PaCLPP (Pa_2_3900), the protease subunit of the mitochondrial matrix CLPPX complex, is found in the grisea mutant. In contrast, transcript levels of the putative chaperone component of the mitochondrial matrix CLPPX complex, PaCLPX, and of the iAAA protease (Pa_3_6030) in the inner mitochondrial membrane, PaIAP, are unchanged. Overall, the transcriptome analysis provides some moderate evidence of an increase in the capacity of components of the mitochondrial protein quality control system in the grisea mutant and perspectives for specific further investigations.

### Secondary Metabolism

During the analyses of the transcriptome data obtained in this comparative study which aimed to elucidate the details of the regulation of cellular copper homeostasis and its particular impact on aging and development, we identified a very strong copper-dependent difference in transcript abundance of a cluster of genes located on chromosome II. The cluster encodes components involved in the synthesis of sterigmatocystin, a precursor of the mycotoxin aflatoxin. All 27 genes annotated to be members of this gene cluster of putative mycotoxin synthesis proteins of *P. anserina* [54] were identified among those transcripts with a strong reduction in abundance in the copper-depletion mutant grisea. These results are well consistent with previously published work from *Aspergillus flavus*, an aflatoxin producer that has been studied in more detail. In one study it was found that moderate addition of metals manganese, copper, cadmium, or chromium to growth media based on corn germ causes significant increases in aflatoxin production [55]. In another study it was found that addition of a mixture of copper, iron, and zinc to the growth medium caused a significant increase in total RNA, biomass, aflP (omtA) transcription, and aflatoxin [56]. One of the four best blast hits for the *A. fumigatus* omtA protein in *P. anserina* is Pa_2_7430 (blast e = 1E−23), which is annotated as putative 8-O-methyltransferase possibly involved in aflatoxin biosynthesis. It is encoded by one of the genes of the aflatoxin gene cluster shown in the present study to be massively down regulated in the grisea mutant strain (Table S10).

We chose *Pa_2_7310* (encoding the putative fatty acid synthase subunit a) to test by qRT-PCR whether the down-regulation of the cluster is due to a direct regulation by GRISEA or due to copper depletion. Since growth of the grisea mutant in copper supplemented growth medium does completely revert the transcript abundance to wild type levels, the down-regulation is clearly due to the copper depletion in the mutant (Fig. 6 C, D). Also the transcription level for *Pa_2_7360*, encoding the putative transcription factor regulating the gene cluster, does – after copper addition to the mutant – have wild type level further supporting the hypothesis that the cluster is regulated by copper availability and not by GRISEA.

Although present in *P. anserina* most likely as the result of horizontal gene transfer from a member of the genus *Aspergillus* [54], the role of this secondary metabolism pathway in *P. anserina* is unclear and the production of aflatoxin or sterigmatocystin is not proven in this species. The presence of such a gene cluster does not necessarily indicate an active production of mycotoxins. In *Aspergillus oryzae*, which is traditionally used in food fermentation industries, the aflatoxin gene cluster is also present, but seems to be inactive due to a loss of function in the *aflJ* gene product [57], [58]. However, the demonstration of a copper-dependent difference in transcript abundance of enzymes controlling this pathway in the current study is certainly an interesting finding calling for further investigations to elucidate the biological role of this pathway and its relationship to aging and development studied in this non-pathogenic fungus.

### Conclusions

The presented differential genome-wide transcriptome analysis is well demonstrating the necessity of a systematic analysis of the consequences of genetic modifications resulting from single point mutations. Starting with a single point mutation in the *Grisea* gene it was expected to identify very specific changes that provide specific conclusions to be drawn for the mechanistic basis of the observed lifespan extension and the impairments in development found in the mutant. As soon as the mutation was identified to be in a copper-regulated transcription factor, it became clear that there are excessive effects to be expected on gene expression in the mutant. These expectations were verified by the current study which identified 1,156 transcripts out of about 10,600 to differ at least 3-fold in abundance. Since the SuperSAGE analysis was performed only with one sample for each strain (but each containing RNA from three individuals) one cannot exclude, that there some of these genes are false positives. However, when comparing the results with highly abundant transcripts (Fig. 1 I and J, or Fig. 6) and rare transcripts (Fig. 1 A and B, C and D), the outcome of the SuperSAGE analysis is well confirmed by the qRT-PCR results. Thus, pooling three different isolates for SuperSAGE analysis seems to provide a realistic picture of the transcript level even of low-abundant transcripts. The analysis is not only relevant for mutants like grisea but also for other mutations or environmental manipulations. Apart from the primary responses (e.g., the lack of a specific enzyme activity) any modification may cause secondary and higher order responses. An analysis just dealing with the immediate responses and affected pathways may miss other important traits that are also significantly contributing to a particular phenotype and process under investigation. With the various available high-throughput technologies and the development of appropriate computational tools, approaches have been developed and are continuously refined that allow to overcome the limitations related to reductionistic approaches to unravel the mechanistic basis of any kind of complex biological process of interest.

## Materials and Methods

### *Podospora anserina* Strains and Cultivation

*P. anserina* wild-type strain ‘s’ [59] and mutant strain grisea [13] were analyzed. Juvenile cultures were derived from mono-nucleate ascospores which were incubated on germination medium as previously described [5]. Pieces of mycelium were subsequently transferred onto cornmeal agar plates (BM medium) and cultivated at 27°C. After two days of growth, the mycelium was scraped off the plate and transferred to Erlenmeyer flasks containing liquid complete medium (CM: 70 mM NH_4_Cl, 7.3 mM KH_2_PO_4_, 6.7 mM KCl, 2 mM MgSO_4_, 1% glucose, 0.2% tryptone, 0.2% yeast extract, 5 µM FeCl_2_, 3.5 µM ZnSO_4_, 6.2 µM MnCl_2_, pH 6.5) and shaken at 27°C in the light according to [60]. Two days later, the mycelium was harvested by filtering it through gaze. The isolated RNAs thus were derived from cultures of the same chronological age. Due to the difference in lifespan of the two strains, biologically the wild-type strains were older than the long-lived grisea mutant strains.

### Isolation of Total RNA

RNA was isolated from fungal mycelium using a CsCl gradient as described previously [5]. Approximately 10 g of mycelium was harvested and ground in liquid nitrogen. The frozen mycelial powder was transferred to 30 ml of pre-warmed (60°C) GTC buffer (5.5 M guanidine thiocyanate, 25 mM sodium citrate, 0.5% N-lauroylsarcosine, 0.2 M β-mercaptoethanol [pH 7.0]), mixed, and incubated for 10 min at 60°C. The sample was centrifuged (10 min, 10,000 rpm, room temperature, Sorvall SS34 rotor), and the supernatant was layered onto 3 ml of CsCl (5.7 M CsCl, 0.1 M EDTA [pH 7.4], refraction index of 1.400) in an ultracentrifuge tube. RNA was centrifuged at 34,000 rpm (18 h, Sorvall TH-641 rotor) at 20°C overnight. The pellet was washed with 70% ethanol and dissolved in dimethyl pyrocarbonate (DMPC)-treated H_2_O.

### Total Protein Extraction

For extraction of whole cell protein, mycelia from different *P. anserina* strains (wild-type ‘s’ and the grisea mutant) was allowed to overgrow a cellophane foil covering a BMM or BMM plate containing 250 µM CuSO_4_ × 5H_2_O, respectively. After incubation for 2 days at 27°C under constant light, the mycelia were transferred into 200 mL liquid CM media or CM media containing 250 µM CuSO_4_ × 5H_2_O for 2 days shaking at 27°C under constant light. The grown mycelia were harvested and subsequently ground in liquid nitrogen. 100 mg of mycelial powder were resuspended in 50 µL extraction buffer (1 mM EDTA, 20 mM HEPES, 50 mM DTT, 1/100 protease inhibitor cocktail [Calbiochem, Nottingham, UK], pH 7.5) and disrupted (Vortex). The sample was centrifuged (11,000 rpm, 10 min, 4°C). The concentration of the supernatant was determined with Roti-Nanoquant (Roth, Karlsruhe, Germany) according to the manufacturer’s instructions.

### Western Blot Analysis

100 µg total protein extracts were fractionated by SDS–PAGE (16% separating gels) according to standard protocols [61]. After electrophoresis, proteins were transferred to PVDF membranes (Immobilon Transfer Membranes, Millipore). Blocking, antibody incubation and washing steps were performed according to the Odyssey Western blot analysis handbook (Licor). As primary antibodies the Anti-PaSOD2 antibody and the Anti-PaPRE3 antibody were used. Anti-PaSOD2 (1∶5.000 dilution) has been raised against a PaSOD2 specific synthetic peptide ([Ac]-CERFLGTSEATKL[OH]; New England Peptide) corresponding to AA 225–236, and Anti-PaPRE3 (1∶2500 dilution) has been raised against a specific synthetic peptide ([H]-LYLPDTDYKVRHEN-[OH]; Sigma) of PaPRE3 (corresponding to the β1 subunit of the 26S proteasome) [39]. In all analyses, secondary antibodies conjugated with IRDye CW 680 or IRDye CW 800 (1∶15,000 dilution; Licor) were used. For detection the Odyssey infrared scanner (Licor) was used.

### ‘In-gel’ SOD Activity Assay

100 µg total protein extracts were loaded on a native polyacrylamide gel (10% separating gels). For staining of gels, nitroblue tetrazolium, riboflavin and TEMED were used as described [62].

### SuperSAGE Analysis

Total RNA from *P. anserina* wild type ‘s’ and the grisea mutant was isolated as described above and subjected to SuperSAGE transcriptome analysis. To decrease differences of gene expression caused by biological subject-to-subject variations, RNA isolated from three independently grown individuals was pooled for each of the two compared strains prior to the SuperSAGE analysis. Pooling of samples is especially important when biological variability is large in relation to the technical variability [63].

SuperSAGE libraries were produced at GenXPro GmbH (Frankfurt, Germany) essentially as described [64], [65]. The resulting 26 bp sequence tags were matched to the *P. anserina* genome database [18] as of October 2011. The *P. anserina* genome database provides only the coding sequence for every known or predicted gene. Sequence tags generated in the SuperSAGE experiment are expected to usually be located downstream of the coding sequence of a transcript molecule. To detect these tags a local database was generated containing every annotated putative coding sequence (CDS) of the *P. anserina* genome database, removing predicted intron sequences from the corresponding coding regions in the genome database. To cover the complete expected transcript sequence of every CDS, each sequence was subsequently extended downstream of the coding region by 500 additional nucleotides. The sequence was taken from the available chromosomal DNA in the database. Tags uniquely matching to one of the resulting sequences were summed up to define a “transcript frequency” of the respective transcript. Tags that could not unambiguously be identified using this local database of expected transcript sequences where additionally searched within EST-sequences contained in the NCBI databases. Statistically significant changes in tag copy number between compared tag libraries were analyzed by calculating a probability (P)-value according to [66] as described in [65]. A gene was only considered to be differentially expressed when showing a calculated P value below 0.01 comparing the tags counted for a gene in the grisea mutant and the wild-type strain. In addition to this calculated P value, the actual number of tags counted in the two different *P. anserina* a strains will be shown as tags per million (tpm) for every gene discussed in this work. When tag counts were zero the “zero substitution value” of 0.05 was assigned before calculation of the fold change value. Calculated fold change values then should be seen as upper or lower limit and are indicated by the prefix “>” or “<”.

### Gene ontology Information

Gene ontology term (GO term) enrichment analysis was performed at GenXPro GmbH (Frankfurt, Germany). Gene ontology (GO) information for the transcripts identified in the SuperSAGE analysis was obtained from www.geneontology.org. P values describing the likelihood for enrichment of GO terms in differentially expressed genes (enrichment p-values) were calculated by the Fisher exact test based on transcripts that were differentially expressed with a p value below 1E-10 when comparing grisea mutant strains to the wild type.

### Quantitative Real-time PCR (qRT-PCR)

Total RNA samples of the following strains and conditions were analyzed: wild type (the three Super-SAGE samples plus two to three additional biological replicates); grisea (the three Super-SAGE samples plus three additional biological replicates). In addition to these samples isolated from cultures grown on medium to which no copper was added, samples from three biological replicates of the wild type and the grisea mutant were isolated from cultures grown on medium supplemented with 250 µM copper sulphate were analyzed. Previously, excess copper has been shown to revert the hypopigmentation phenotype of the mutant grisea and leads to wild type-like instability of the mtDNA [14], [67].

Reverse transcription of 1.5 µg total RNA was performed using the iScript kit (Bio-Rad) and the primers summarized in Table S11. After reverse transcription the cDNA was diluted 1∶7.5 and 2–4 µl were used for the PCR (iQ SybrGreen SuperMix, Bio-Rad). For each gene, the PCR efficiency was determined according to [68]. The relative expression level (normalized to the level of the porin transcript) was calculated according to the following formula:

Relative expression = (E(porin)^∧^CP(porin))/(E(target gene)^∧^CP(target gene)) with: E = PCR efficiency of the respective primer pair and CP = crossing point for each transcript.

## Supporting Information

Table S1 **GO term enrichment analysis.**
(DOCX)

Table S2 **Selected genes of** ***P. anserina*** **and their expression level comparing grisea mutant strain to the wild type.**
(DOCX)

Table S3 **Transcripts encoding proteins of RIA iron transport.**
(DOCX)

Table S4 **Transcripts of siderophore iron transport proteins.**
(DOCX)

Table S5 **Transcripts of proteins involved in catabolic processes.**
(DOCX)

Table S6 **Transcripts of proteins of the respiratory chain.**
(DOCX)

Table S7 **Transcripts of proteins involved in ROS detoxification.**
(DOCX)

Table S8 **Transcripts of melanin synthesis proteins.**
(DOCX)

Table S9 **Transcripts of proteins involved in mitochondrial protein quality control.**
(DOCX)

Table S10 **Transcripts of proteins of the secondary metabolism.**
(DOCX)

Table S11 **Summary of primer sequences used in qRT-PCR analysis of selected genes.**
(DOCX)
