# Supplementary material for: A Differential Genome-Wide Transcriptome Analysis: Impact of Cellular Copper on Complex Biological Processes like Aging and Development
Source: PLoS One. 2012 Nov 12;7(11):e49292. doi: 10.1371/journal.pone.0049292 (PMC3495915; doi:10.1371/journal.pone.0049292)
Supplement: Table S1 — GO term enrichment analysis. (DOCX) [file pone.0049292.s001.docx]

**Table S1. GO term enrichment analysis.**

| **GO-Term** | **Transcripts** | **Differential** | **Up** | **Down** | **Enrichment p value** | **Description** |
| --- | --- | --- | --- | --- | --- | --- |
| GO:0005739 | 672 | 282 | 47 | 235 | 3.70E-40 | mitochondrion |
| GO:0044429 | 361 | 166 | 23 | 143 | 1.29E-28 | mitochondrial part |
| GO:0007005 | 103 | 63 | 2 | 61 | 2.83E-19 | mitochondrion organization |
| GO:0032543 | 53 | 40 | 0 | 40 | 1.47E-17 | mitochondrial translation |
| GO:0005759 | 120 | 64 | 12 | 52 | 2.03E-15 | mitochondrial matrix |
| GO:0005740 | 255 | 106 | 12 | 94 | 1.13E-14 | mitochondrial envelope |
| GO:0042180 | 466 | 165 | 65 | 100 | 1.97E-14 | cellular ketone metabolic process |
| GO:0031966 | 238 | 98 | 11 | 87 | 2.44E-13 | mitochondrial membrane |
| GO:0019752 | 454 | 158 | 64 | 94 | 4.06E-13 | carboxylic acid metabolic process |
| GO:0043436 | 454 | 158 | 64 | 94 | 4.06E-13 | oxoacid metabolic process |
| GO:0055114 | 762 | 237 | 98 | 139 | 4.96E-13 | oxidation-reduction process |
| GO:0006082 | 463 | 160 | 64 | 96 | 5.57E-13 | organic acid metabolic process |
| GO:0005743 | 166 | 75 | 5 | 70 | 6.97E-13 | mitochondrial inner membrane |
| GO:0019866 | 174 | 76 | 6 | 70 | 4.03E-12 | organelle inner membrane |
| GO:0000313 | 29 | 23 | 0 | 23 | 2.18E-11 | organellar ribosome |
| GO:0005761 | 29 | 23 | 0 | 23 | 2.18E-11 | mitochondrial ribosome |
| GO:0016491 | 716 | 219 | 94 | 125 | 2.93E-11 | oxidoreductase activity |
| GO:0005840 | 229 | 90 | 27 | 63 | 5.18E-11 | ribosome |
| GO:0031967 | 337 | 118 | 17 | 101 | 3.27E-10 | organelle envelope |
| GO:0031975 | 348 | 120 | 18 | 102 | 6.83E-10 | envelope |
| GO:0006520 | 286 | 103 | 36 | 67 | 7.91E-10 | cellular amino acid metabolic process |
| GO:0044106 | 290 | 103 | 36 | 67 | 1.92E-09 | cellular amine metabolic process |
| GO:0006091 | 214 | 81 | 15 | 66 | 4.04E-09 | generation of precursor metabolites and energy |
| GO:0044283 | 404 | 132 | 47 | 85 | 4.92E-09 | small molecule biosynthetic process |
| GO:0003735 | 194 | 75 | 24 | 51 | 5.25E-09 | structural constituent of ribosome |
| GO:0000315 | 16 | 14 | 0 | 14 | 2.04E-08 | organellar large ribosomal subunit |
| GO:0005762 | 16 | 14 | 0 | 14 | 2.04E-08 | mitochondrial large ribosomal subunit |
| GO:0044444 | 2505 | 608 | 220 | 388 | 2.61E-08 | cytoplasmic part |
| GO:0048037 | 336 | 111 | 50 | 61 | 4.64E-08 | cofactor binding |
| GO:0009308 | 344 | 113 | 40 | 73 | 4.93E-08 | amine metabolic process |
| GO:0044281 | 1233 | 326 | 133 | 193 | 5.72E-08 | small molecule metabolic process |
| GO:0006732 | 131 | 54 | 14 | 40 | 6.25E-08 | coenzyme metabolic process |
| GO:0046394 | 237 | 84 | 32 | 52 | 7.08E-08 | carboxylic acid biosynthetic process |
| GO:0016053 | 239 | 84 | 32 | 52 | 1.09E-07 | organic acid biosynthetic process |
| GO:0051186 | 176 | 66 | 17 | 49 | 1.72E-07 | cofactor metabolic process |
| GO:0044271 | 340 | 110 | 33 | 77 | 1.83E-07 | cellular nitrogen compound biosynthetic process |
| GO:0022900 | 77 | 36 | 9 | 27 | 2.55E-07 | electron transport chain |
| GO:0016651 | 45 | 25 | 4 | 21 | 2.86E-07 | oxidoreductase activity, acting on NADH or NADPH |
| GO:0008652 | 164 | 58 | 18 | 40 | 8.32E-06 | cellular amino acid biosynthetic process |
| GO:0009309 | 164 | 58 | 18 | 40 | 8.32E-06 | amine biosynthetic process |

*Podospora* genes differentially expressed with a high significance level (p < 1E-10) analyzed for the distribution of Gene Ontology (GO) terms assigned to them. Listed are the most significantly enriched GO-terms (enrichment p value < 1E-05) among genes differentially expressed in the grisea mutant strain, sorted by enrichment p value.
GO term: the identification number of the GO term. Transcripts: the number of all genes measured that are assigned to the respective GO term. Differential: the number of genes differentially expressed. Up/Down: the number of genes with increased or decreased amount of transcript in the grisea mutant strain compared to the wild type. Enrichment p value: the significance level of GO term enrichment. Description: description of the respective GO term.
