# Supplementary material for: A Differential Genome-Wide Transcriptome Analysis: Impact of Cellular Copper on Complex Biological Processes like Aging and Development
Source: PLoS One. 2012 Nov 12;7(11):e49292. doi: 10.1371/journal.pone.0049292 (PMC3495915; doi:10.1371/journal.pone.0049292)
Supplement: Table S2 — Selected genes of P. anserina and their expression level comparing grisea mutant strain to the wild type. (DOCX) [file pone.0049292.s002.docx]

**Table S2. Selected genes of *P. anserina* and their expression level comparing grisea mutant strain to the wild type.**

| **PaNo** | **Annotation in the *P. anserina* genome database/ Protein description** | **FC (grisea/WT)** | **Tpm (wt)** | **Tpm (grisea)** | **P value** |
| --- | --- | --- | --- | --- | --- |
| *Pa_1_16400* | Putative copper transport protein/ *PaCtr1*, target gene of GRISEA | 0.10 | 85.70 | 8.56 | 0.000 |
| *Pa_4_4770* | Low-affinity copper transport protein encoded by the ctr2 gene/ *PaCtr2*, target gene of Grisea encoding a high affinity copper transporter | 0.06 | 15.14 | 0.98 | 0.000 |
| *Pa_3_10440* | Copper transporter encoded by the Pactr3 gene/ *PaCtr3*, target gene of GRISEA, encoding high affinity copper transporter | < 0.04 | 1.41 | < 0.05 | 0.001 |
| *Pa_1_4220* | Putative protein of unknown function/ low affinity copper transporter | 1.49 | 91.73 | 136.22 | 0.000 |

PaNo: accession number in the *P. anserina* genome database as found by the blast search. FC: the difference of expression comparing grisea mutant strain to wild type (fold change). Tpm: the number of transcript molecules normalized as tags per million. P value: the significance level of differential expression comparing the *Podospora* grisea mutant strain to the wild type. ‘Protein description’ entries indicate conclusions from the current transcriptome and qRT-PCR analysis.
