# Supplementary material for: A Differential Genome-Wide Transcriptome Analysis: Impact of Cellular Copper on Complex Biological Processes like Aging and Development
Source: PLoS One. 2012 Nov 12;7(11):e49292. doi: 10.1371/journal.pone.0049292 (PMC3495915; doi:10.1371/journal.pone.0049292)
Supplement: Table S3 — Transcripts encoding proteins of RIA iron transport. (DOCX) [file pone.0049292.s003.docx]

**Table S3. Transcripts encoding proteins of RIA iron transport.**

| ***S. cerevisiae* RIA type iron transport protein (UniProt-Acc.)** | **E-value of blast search result** | **PaNo** | **Annotation in the *P. anserina* genome database/ Protein description** | **FC (gr/wt)** | **Tpm  (wt)** | **Tpm (grisea)** | **P value** |
| --- | --- | --- | --- | --- | --- | --- | --- |
| **RIA (reductive iron assimilation): FRE1, FRE2, FET3, FTR1** | |  |  |  |  |  |  |
| Ferric/cupric reductase transmembrane components Fre1 (P32791), Fre2 (P36033) | 9.0E-32, 8.0E-30 | Pa_5_11970 | Putative ferric reductase transmembrane component/ *PaFre1*, putative target gene of GRISEA | 0.00 | 26.68 | <0.05 | 0.000 |
|  | 9.0E-29, 2.0E-28 | Pa_7_5660 | Putative ferric reductase transmembrane component | 0.68 | 179.60 | 122.61 | 0.000 |
|  | 1.0E-23, 3.0E-27 | Pa_5_10230 | Putative ferric reductase transmembrane component | - | - | - | - |
|  | 5.0E-22, 2.0E-23 | Pa_1_19630 | Putative ferric reductase transmembrane component | 0.44 | 45.41 | 20.20 | 0.000 |
|  | 2.0E-21, 1.0E-17 | Pa_1_16410 | Putative ferric reductase transmembrane component | 0.21 | 53.83 | 12.06 | 0.000 |
|  | 2.0E-16, 6.0E-12 | Pa_1_19550 | Putative ferric reductase transmembrane component | 2.57 | 25.02 | 64.25 | 0.000 |
| Plasma membrane iron permease Ftr1 (P40088) | 2.0E-55 | Pa_6_4210 | Putative plasma membrane iron permease | (ns) | 0.90 | 0.28 | 0.144 |
| Iron transport multicopper oxidase Fet3(P38993) | 1.0E-130 | Pa_6_4220 | Putative precursor of Iron transport multicopper oxidase FET3 | (ns) | 0.38 | < 0.05 | 0.149 |
|  | 3.0E-78 | Pa_2_530 | Putative iron transport multicopper oxidase FET3 | 0.08 | 16.29 | 1.26 | 0.000 |
|  | 3.0E-56 | Pa_6_2550 | Putative multicopper oxidase | (ns) | 1.67 | 1.68 | 0.972 |
|  | 7.0E-47 | Pa_5_1200 | Laccase-2 precursor | (ns) | 0.26 | 0.14 | 0.693 |

‘E-value of blast search result’: the e-values of the results of a blast search using the sequence of the *S. cerevisiae* proteins given in the first column (UniProtID given in brackets) against the *Podospora anserina* genome database. PaNo: accession number in the *P. anserina* genome database as found by the blast search. FC: the difference of expression comparing grisea mutant strain to wild type (fold change). Tpm: the number of transcript molecules normalized as tags per million. P value: the significance level of differential expression comparing the *Podospora* grisea mutant strain to the wild type. (ns) indicates a non-significant differential expression (p>0.01). ‘Protein description’ entries indicate conclusions from the current transcriptome and qRT-PCR analysis.
