# Supplementary material for: A Differential Genome-Wide Transcriptome Analysis: Impact of Cellular Copper on Complex Biological Processes like Aging and Development
Source: PLoS One. 2012 Nov 12;7(11):e49292. doi: 10.1371/journal.pone.0049292 (PMC3495915; doi:10.1371/journal.pone.0049292)
Supplement: Table S4 — Transcripts of siderophore iron transport proteins. (DOCX) [file pone.0049292.s004.docx]

**Table S4. Transcripts of siderophore iron transport proteins.**

| **PaNo** | **Annotation in the *P. anserina* genome database** | **FC (grisea/wt)** | **Tpm (wt)** | **Tpm (grisea)** | **P value** | **Blast e value (putative homolog of *A. fumigatus*)** |
| --- | --- | --- | --- | --- | --- | --- |
| Pa_4_4430 | Putative L-ornithine 5-monooxygenase | 2.49 | 11.55 | 28.76 | 0.000 | e=1E-119 (AfSidA, Q5SE95) |
| Pa_5_4760 | Putative protein of unknown function | (ns) | 13.21 | 14.45 | 0.520 | e=1E-107 (AfSidF, Q4WF55) |
| Pa_3_11200 | Putative HC-toxin synthetase | (ns) | 4.23 | 6.45 | 0.064 | e=0.0 (nonribosomal peptide synthetase AfSidD, Q4WF53) |
| Pa_4_4640 | Putative peptide synthase | 0.48 | 88.39 | 42.37 | 0.000 | e=0.0 (nonribosomal peptide synthetase AfSidD, Q4WF53) |
| Pa_5_1070 | Putative protein of unknown function | (ns) | 27.58 | 23.57 | 0.127 | e=5E-92 (nonribosomal peptide synthetase AfSidD, Q4WF53) |
| - | - | - | - | - | - | (no blast hit of e < 1.7 for AfSidG, Q4WF30) |
| Pa_4_4440 | Putative peptide synthetase | 3.06 | 5.77 | 17.68 | 0.000 | e=0.0 (AfSidC, B0XP99) |
| Pa_4_4640 | Putative peptide synthase | 0.48 | 88.39 | 42.37 | 0.000 | e=1E-121 (AfSidC, B0XP99) |
| Pa_2_7870 | Putative linear gramicidin synthetase | 0.02 | 964.47 | 16.69 | 0.000 | e=1E-119 (AfSidC, B0XP99) |
| Pa_7_120 | Putative siderophore iron transporter | 2.34 | 62.99 | 147,04 | 0.000 | e=6E-88 (AfMfs, putative MirB-like siderophore transporter, B0XZP9) |
| Pa_5_3540 | Putative siderophore iron transporter | 0.73 | 607.18 | 443.72 | 0.000 | e=1E-117 (AfMirB, siderophore transporter, Q4WF31) |
| Pa_6_1720 | Putative siderophore regulation protein | 0.47 | 63.63 | 30.16 | 0.000 | e = 2E-40 (AfSreA, siderophore transcription factor, Q4WV91) |
| Pa_5_4490 | Putative siderophore iron transporter 1 | (ns) | 3.59 | 2.81 | 0.407 | e=0.0 (AfSit1, putative siderochrome-iron transporter, Q4WGS5) |
| Pa_7_6950 | Putative siderophore iron transporter | 1.60 | 18.47 | 29.60 | 0.000 | e=1E-151 (AfMirC, siderochrome-iron transporter MirC, Q4WHE1) |
| Pa_3_1250 | Putative protein of unknown function | 10.93 | 0.26 | 2.81 | 0.000 | e=2E-38 (AfEstB, putative siderophore esterase IroE-like, Q4WF29) |
| Pa_2_8330 | Putative protein of unknown function | (ns) | 0.13 | < 0.05 | 0.546 | e=1E-37 (AfEstB, putative siderophore esterase IroE-like, Q4WF29) |
| Pa_4_1300 | Putative protein of unknown function | (ns) | 23.35 | 28.48 | 0.051 | e=8E-30 (AfEstB, putative siderophore esterase IroE-like, Q4WF29) |

PaNo: accession number in the *P. anserina* genome database as found by the blast search. FC: the difference of expression comparing grisea mutant strain to wild type (fold change). Tpm: the number of transcript molecules normalized as tags per million. P value: the significance level of differential expression comparing the *Podospora* grisea mutant strain to the wild type. (ns) indicates a non-significant differential expression (p>0.01).
