# Supplementary material for: A Differential Genome-Wide Transcriptome Analysis: Impact of Cellular Copper on Complex Biological Processes like Aging and Development
Source: PLoS One. 2012 Nov 12;7(11):e49292. doi: 10.1371/journal.pone.0049292 (PMC3495915; doi:10.1371/journal.pone.0049292)
Supplement: Table S5 — Transcripts of proteins involved in catabolic processes. (DOCX) [file pone.0049292.s005.docx]

**Table S5. Transcripts of proteins involved in catabolic processes.**

| **PaNo** | **Annotation at the *P. anserina* genome database** | **FC (grisea/wt)** | **Tpm (wt)** | **Tpm (grisea)** | **P value** |
| --- | --- | --- | --- | --- | --- |
| Pa_1_15110 | Putative glucokinase | 3.13 | 129.83 | 406.96 | 0.000 |
| Pa_6_10000 | Putative mitochondrial precursor of 2-methylcitrate synthase | 0.08 | 41.82 | 3.23 | 0.000 |
| Pa_2_12390 | Putative aconitate hydratase, mitochondrial precursor | 3.09 | 20.42 | 63.20 | 0.000 |
| Pa_1_19320 | Putative mitochondrial fumarate hydratase precursor | 4.33 | 167.29 | 724.15 | 0.000 |

PaNo: accession number in the *P. anserina* genome database as found by the blast search. FC: the difference of expression comparing grisea mutant strain to wild type (fold change). Tpm: the number of transcript molecules normalized as tags per million. P value: the significance level of differential expression comparing the *Podospora* grisea mutant strain to the wild type.
