# Supplementary material for: A Differential Genome-Wide Transcriptome Analysis: Impact of Cellular Copper on Complex Biological Processes like Aging and Development
Source: PLoS One. 2012 Nov 12;7(11):e49292. doi: 10.1371/journal.pone.0049292 (PMC3495915; doi:10.1371/journal.pone.0049292)
Supplement: Table S6 — Transcripts of proteins of the respiratory chain. (DOCX) [file pone.0049292.s006.docx]

**Table S6. Transcripts of proteins of the respiratory chain.**

| **Complex of the respiratory chain** | **PaNo** | **Annotation at the *P. anserina* genome database** | **FC (grisea/wt)** | **Tpm (wt)** | **Tpm (grisea)** | **P value** |
| --- | --- | --- | --- | --- | --- | --- |
|  | Pa_3_1710 | AOX1, alternative oxidase mitochondrial precursor encoded by the AOX gene | 19.81 | 100.71 | 1994.70 | 0.000 |
| I | Pa_mito_nad6 | NADH-ubiquinone oxidoreductase chain 6 | 2.05 | 51.44 | 105.47 | 0.000 |
| I | Pa_5_9540 | Putative NADH-ubiquinone oxidoreductase 21.3 kDa subunit | 0.89 | 535.22 | 477.81 | 0.000 |
| II | Pa_1_8920 | Putative mitochondrial succinate dehydrogenase [ubiquinone] iron-sulfur subunit precursor | 2.44 | 184.61 | 450.31 | 0.000 |
| II | Pa_2_7010 | Putative succinate dehydrogenase cytochrome B subunit, mitochondrial precursor | 2.03 | 397.05 | 804.25 | 0.000 |
| III | Pa_6_240 | Putative mitochondrial precursor of cytochrome b-c1 complex subunit Rieske | 2.11 | 602.44 | 1273.22 | 0.000 |
| III | Pa_2_12750 | Putative cytochrome b-c1 complex subunit 8 precursor | 2.62 | 435.28 | 1139.67 | 0.000 |
| III | Pa_6_4730 | Putative mitochondrial precursor of Cytochrome b-c1 complex subunit 2 | 1.55 | 1295.96 | 2013.49 | 0.000 |
| IV | Pa_3_8970 | Putative cytochrome c oxidase polypeptide VIb | 2.41 | 9.24 | 22.31 | 0.000 |
| IV | Pa_2_11540 | Putative cytochrome c oxidase polypeptide VI, mitochondrial precursor | 1.82 | 264.40 | 482.16 | 0.000 |
| IV | Pa_mito_cox1 | Cytochrome c oxidase subunit 1 | 1.29 | 10.90 | 14.03 | 0.087 |
| IV | Pa_mito_cox2 | Cytochrome c oxidase subunit 2 | 0.46 | 17.19 | 7.86 | 0.000 |
| IV | Pa_mito_cox3 | Cytochrome c oxidase subunit 3 | 0.63 | 44.64 | 28.06 | 0.000 |
| V | Pa_1_13400 | Putative mitochondrial ATPase complex subunit ATP10 | 3.43 | 28.74 | 98.48 | 0.000 |
| V | Pa_7_2170 | Putative mitochondrial ATP synthase delta chain precursor | 1.49 | 1046.57 | 1562.48 | 0.000 |

PaNo: accession number in the *P. anserina* genome database as found by the blast search. FC: the difference of expression comparing grisea mutant strain to wild type (fold change). Tpm: the number of transcript molecules normalized as tags per million. P value: the significance level of differential expression comparing the *Podospora* grisea mutant strain to the wild type.
