# Supplementary material for: A Differential Genome-Wide Transcriptome Analysis: Impact of Cellular Copper on Complex Biological Processes like Aging and Development
Source: PLoS One. 2012 Nov 12;7(11):e49292. doi: 10.1371/journal.pone.0049292 (PMC3495915; doi:10.1371/journal.pone.0049292)
Supplement: Table S7 — Transcripts of proteins involved in ROS detoxification. (DOCX) [file pone.0049292.s007.docx]

**Table S7. Transcripts of proteins involved in ROS detoxification.**

| **PaNo** | **Annotation at the *P. anserina* genome database/ Protein description** | **FC (grisea/wt)** | **Tpm (wt)** | **Tpm (grisea)** | **P value** |
| --- | --- | --- | --- | --- | --- |
| Pa_1_17400 | Putative superoxide dismutase [Cu-Zn]/ PaSOD1 | 1.05 | 1935.73 | 2037.62 | 0.000 |
| Pa_2_4460 | Putative superoxide dismutase [Mn], mitochondrial precursor /PaSOD2 | (ns) | 69.79 | 68.74 | 0.809 |
| Pa_5_1740 | Putative superoxide dismutase [Mn] /PaSOD3 | 1.97 | 21.04 | 41.52 | 0.000 |
| Pa_1_10620 | Putative mitochondrial SSU ribosomal protein S26 precursor**/** Putative superoxide dismutase PaSOD4 | 3.28 | 41.69 | 136.92 | 0.000 |
| Pa_6_11240 | PaCAT2 catalase-peroxydase encoded by the PaCat2 gene/ PaCAT2 | 0.42 | 421.94 | 176.76 | 0.000 |
| Pa_1_6960 | Putative mitochondrial cytochrome c peroxidase precursor | 2.64 | 245.93 | 648.53 | 0.000 |
| Pa_7_1610 | CATB large catalase encoded by the catB gene/ PaCATB | 1.86 | 166.90 | 311.01 | 0.000 |
| Pa_7_4240 | CATA large catalase encoded by the catA gene | (ns) | 1.28 | 2.10 | 0.226 |
| Pa_5_4690 | Putative peroxiredoxin | 1.63 | 483.77 | 790.36 | 0.000 |
| Pa_1_14500 | Putative peroxisomal prx5-like peroxiredoxin | 3.14 | 139.96 | 439.65 | 0.000 |
| Pa_5_8240 | Putative peroxisomal membrane protein (PMP20) prx5-like peroxiredoxin/ PaPRX1**,** mitochondrially located and differentially expressed during aging [41,43] | 0.59 | 319.8 | 189.2 | 0.000 |
| Pa_1_6960 | Putative mitochondrial cytochrome c peroxidase precursor | 2.64 | 245.93 | 648.53 | 0.000 |
| Pa_2_8460 | Putative glutathion peroxidase | 2.86 | 29.12 | 83.19 | 0.000 |
| Pa_2_10 | Putative peroxidase | (ns) | 0.13 | < 0.05 | 0.546 |
| Pa_2_7880 | Putative SAM-dependent O-methyltransferase encoded by the PaMTH1 gene/ PaMTH1 | 0.06 | 5271.72 | 334.30 | 0.00 |

PaNo: accession number in the *P. anserina* genome database as found by the blast search. FC: the difference of expression comparing grisea mutant strain to wild type (fold change). Tpm: the number of transcript molecules normalized as tags per million. P value: the significance level of differential expression comparing the *Podospora* grisea mutant strain to the wild type. (ns) indicates a non-significant differential expression (p>0.01). ‘Protein description’ entries indicate conclusions from the current transcriptome and qRT-PCR analysis.
