# Supplementary material for: A Differential Genome-Wide Transcriptome Analysis: Impact of Cellular Copper on Complex Biological Processes like Aging and Development
Source: PLoS One. 2012 Nov 12;7(11):e49292. doi: 10.1371/journal.pone.0049292 (PMC3495915; doi:10.1371/journal.pone.0049292)
Supplement: Table S8 — Transcripts of melanin synthesis proteins. (DOCX) [file pone.0049292.s008.docx]

**Table S8. Transcripts of melanin synthesis proteins.**

| **PaNo** | **Annotation in the *P. anserina* genome database** | **FC (grisea/wt)** | **Tpm (wt)** | **Tpm (grisea)** | **P value** |
| --- | --- | --- | --- | --- | --- |
| Pa_2_510 | PaPKS1 melanin biosynthesis polyketide synthase encoded by the PaPKS1 gene | 0.38 | 19.88 | 7.58 | 0.000 |
| Pa_5_11880 | Putative tetrahydroxy­naphthalene reductase | 0.11 | 40.67 | 4.35 | 0.000 |
| Pa_7_11610 | Putative scytalone dehydratase | 0.30 | 7.44 | 2.24 | 0.000 |
| Pa_5_1990 | Putative hydroxynaphthalene reductase | 0.24 | 31.56 | 7.44 | 0.000 |
| Pa_1_15470 | Putative laccase precursor | 0.23 | 79.28 | 18.52 | 0.000 |
| Pa_5_1200 | Laccase-2 precursor | (ns) | 0.26 | 0.14 | 0.693 |
| Pa_5_4140 | Putative laccase precursor | 0.04 | 86.98 | 3.65 | 0.000 |
| Pa_5_4660 | Putative laccase precursor | - | - | - | - |
| Pa_5_9860 | Putative laccase precursor | 6.99 | 2.95 | 20.62 | 0.000 |
| Pa_6_10630 | Putative laccase precursor | - | - | - | - |
| Pa_6_7880 | Putative laccase precursor | 0.37 | 5656.32 | 2070.17 | 0.000 |
| Pa_7_3560 | Putative laccase precursor | 1.78 | 1.03 | 1.82 | 0.201 |
| Pa_7_4200 | Putative laccase precursor | - | - | - | - |

PaNo: accession number in the *P. anserina* genome database as found by the blast search. FC: the difference of expression comparing grisea mutant strain to wild type (fold change). Tpm: the number of transcript molecules normalized as tags per million. P value: the significance level of differential expression comparing the *Podospora* grisea mutant strain to the wild type. (ns) indicates a non-significant differential expression (p>0.01).
