# Supplementary material for: A Differential Genome-Wide Transcriptome Analysis: Impact of Cellular Copper on Complex Biological Processes like Aging and Development
Source: PLoS One. 2012 Nov 12;7(11):e49292. doi: 10.1371/journal.pone.0049292 (PMC3495915; doi:10.1371/journal.pone.0049292)
Supplement: Table S9 — Transcripts of proteins involved in mitochondrial protein quality control. (DOCX) [file pone.0049292.s009.docx]

**Table S9. Transcripts of proteins involved in mitochondrial protein quality control.**

| **PaNo** | **Annotation in the *P. anserina* genome database** | **FC (grisea/wt)** | **Tpm (wt)** | **Tpm (grisea)** | **P value** |
| --- | --- | --- | --- | --- | --- |
| Pa_3_4170 | Putative Lon protease, mitochondrial precursor | 1.68 | 363.82 | 612.06 | 0.000 |
| Pa_6_5750 | Putative mitochondrial precursor of Heat shock protein 60 | 2.52 | 431.17 | 1085.94 | 0.000 |
| Pa_3_6030 | Putative protein similar to intermembrane space AAA protease IAP-1 of *Neurospora crassa* | (ns) | 76.97 | 67.20 | 0.027 |
| Pa_6_5590 | Putative chaperone MCX1 | 1.08 | 24.12 | 26.09 | 0.446 |
| Pa_2_3900 | Putative ATP-dependent Clp protease proteolytic subunit | 1.82 | 67.02 | 125.27 | 0.000 |
| Pa_2_5010 | Putative mitochondrial respiratory chain complexes assembly protein | 4.88 | 66.20 | 323.21 | 0.000 |
| Pa_6_2570 | Putative precursor of mitochondrial heat shock protein SSC1 | 4.43 | 2.69 | 11.92 | 0.000 |

PaNo: accession number in the *P. anserina* genome database as found by the blast search. FC: the difference of expression comparing grisea mutant strain to wild type (fold change). Tpm: the number of transcript molecules normalized as tags per million. P value: the significance level of differential expression comparing the *Podospora* grisea mutant strain to the wild type. (ns) indicates a non-significant differential expression (p>0.01).
