# Supplementary material for: A Differential Genome-Wide Transcriptome Analysis: Impact of Cellular Copper on Complex Biological Processes like Aging and Development
Source: PLoS One. 2012 Nov 12;7(11):e49292. doi: 10.1371/journal.pone.0049292 (PMC3495915; doi:10.1371/journal.pone.0049292)
Supplement: Table S10 — Transcripts of proteins of the secondary metabolism. (DOCX) [file pone.0049292.s010.docx]

**Table S10. Transcripts of proteins of the secondary metabolism.**

| **PaNo** | **Annotation in the *P. anserina* genome database** | **FC (grisea/wt)** | **Tpm (wt)** | **Tpm (grisea)** | **P value** |
| --- | --- | --- | --- | --- | --- |
| Pa_2_7210 | Putative esterase paralogous to A. nidulans stcI, possibly a member of the aflatoxin biosynthesis cluster | 0.04 | 10.65 | 0.42 | 0.000 |
| Pa_2_7220 | Putative FAD-containing monooxygenase involved in aflatoxin biosynthesis orthologous to *A. nidulans* stcW, member of the aflatoxin cluster | 0.00 | 177.42 | 0.70 | 0.000 |
| Pa_2_7230 | Putative reductase involved in aflatoxin biosynthesis orthologous to *A. nidulans* stcV, member of the aflatoxin cluster | 0.01 | 2674.79 | 15.99 | 0.000 |
| Pa_2_7240 | Putative reductase involved in aflatoxin biosynthesis orthologous to *A. nidulans* stcU, member of the aflatoxin cluster | 0.00 | 1359.59 | 0.14 | 0.000 |
| Pa_2_7250 | Putative dehydrogenase/reductase involved in aflatoxin biosynthesis orthologous to *A. nidulans* stcT, member of the aflatoxin cluster | 0.00 | 159.20 | 0.14 | 0.000 |
| Pa_2_7255 | Putative protein of unknown function possibly involved in aflatoxin biosynthesis, member of the aflatoxin cluster | 0.02 | 142.66 | 2.67 | 0.000 |
| Pa_2_7260 | Putative cytochrome P450 E-class, group I involved in aflatoxin biosynthesis orthologous to *A. nidulans* stcS, member of the aflatoxin cluster | 0.00 | 186.91 | 0.14 | 0.000 |
| Pa_2_7270 | Putative GMC oxidoreductase possibly involved in aflatoxin biosynthesis, member of the aflatoxin cluster | 0.00 | 101.60 | < 0.05 | 0.000 |
| Pa_2_7280 | Putative protein of unknown possibly function involved in aflatoxin biosynthesis, member of the aflatoxin cluster | 0.00 | 122.26 | < 0.05 | 0.000 |
| Pa_2_7290 | Putative cytochrome P450 pisatin demethylase-like involved in aflatoxin biosynthesis orthologous to *A. nidulans* stcL, member of the aflatoxin cluster | 0.03 | 89.93 | 2.81 | 0.000 |
| Pa_2_7300 | Putative fatty acid synthase subunit beta involved in aflatoxin biosynthesis orthologous to *A. nidulans* stcK, member of the aflatoxin cluster | 0.00 | 13.73 | < 0.05 | 0.000 |
| Pa_2_7310 | Putative fatty acid synthase subunit alpha involved in aflatoxin biosynthesis orthologous to *A. nidulans* stcJ, member of the aflatoxin cluster | 0.01 | 196.28 | 1.96 | 0.000 |
| Pa_2_7320 | Putative lipase/esterase involved in aflatoxin biosynthesis orthologous to *A. nidulans* stcI, member of the aflatoxin cluster | 0.00 | 191.53 | < 0.05 | 0.000 |
| Pa_2_7325 | Putative protein of unknown function possibly involved in aflatoxin biosynthesis, member of the aflatoxin cluster | 0.00 | 248.24 | < 0.05 | 0.000 |
| Pa_2_7330 | Putative short-chain dehydrogenase/reductase possibly involved in aflatoxin biosynthesis, member of the aflatoxin cluster | 0.01 | 1015.01 | 5.19 | 0.000 |
| Pa_2_7340 | Putative cytochrome P450 pisatin demethylase-like involved in aflatoxin biosynthesis orthologous to *A. nidulans* stcF, member of the aflatoxin cluster | 0.00 | 109.43 | 0.28 | 0.000 |
| Pa_2_7350 | Putative protein of unknown function involved in aflatoxin biosynthesis rthologous to *A. nidulans* AflJ, member of the aflatoxin cluster | 0.02 | 349.20 | 7.01 | 0.000 |
| Pa_2_7360 | Putative transcription factor orthologous to *A. nidulans* aflR involved in the regulation of the aflatoxin cluster, member of the aflatoxin cluster | 0.01 | 62.60 | 0.84 | 0.000 |
| Pa_2_7370 | Putative ketoreductase involved in aflatoxin biosynthesis orthologous to *A. nidulans* stcE, member of the aflatoxin cluster | 0.01 | 533.80 | 3.79 | 0.000 |
| Pa_2_7380 | Putative protein of unknown function possibly involved in aflatoxin biosynthesis, member of the aflatoxin cluster | 0.14 | 471.07 | 64.25 | 0.000 |
| Pa_2_7390 | Putative aromatic peroxygenase involved in aflatoxin biosynthesis orthologous to *A. nidulans* stcC, member of the aflatoxin cluster | 0.03 | 17.83 | 0.56 | 0.000 |
| Pa_2_7400 | Putative cytochrome P450 E-class, group I involved in aflatoxin biosynthesis orthologous to *A. nidulans* stcB, member of the aflatoxin cluster | 0.00 | 96.06 | < 0.05 | 0.000 |
| Pa_2_7410 | Putative polyketide synthase involved in aflatoxin biosynthesis orthologous to *A. nidulans* stcA, member of the aflatoxin cluster | 0.00 | 379.22 | < 0.05 | 0.000 |
| Pa_2_7420 | Putative NAD(P)-binding protein involved in aflatoxin biosynthesis orthologous to *A. nidulans* stcO, member of the aflatoxin cluster | 0.00 | 77.10 | < 0.05 | 0.000 |
| Pa_2_7430 | Putative 8-O-methyltransferase possibly involved in aflatoxin biosynthesis, member of the aflatoxin cluster | 0.00 | 510.33 | 0.14 | 0.000 |
| Pa_2_7440 | Putative NAD(P)-binding protein involved in aflatoxin biosynthesis orthologous to *A. nidulans* stcQ, member of the aflatoxin cluster | 0.00 | 425.02 | 0.56 | 0.000 |
| Pa_2_7450 | Putative protein of unknown function possibly involved in aflatoxin biosynthesis, member of the aflatoxin cluster | 0.00 | 318.02 | < 0.05 | 0.000 |

PaNo: accession number in the *P. anserina* genome database as found by the blast search. FC: the difference of expression comparing grisea mutant strain to wild type (fold change). Tpm: the number of transcript molecules normalized as tags per million. P value: the significance level of differential expression comparing the *Podospora* grisea mutant strain to the wild type. ‘Protein description’ entries indicate conclusions from the current transcriptome and qRT-PCR analysis.
