# Supplementary material for: A Differential Genome-Wide Transcriptome Analysis: Impact of Cellular Copper on Complex Biological Processes like Aging and Development
Source: PLoS One. 2012 Nov 12;7(11):e49292. doi: 10.1371/journal.pone.0049292 (PMC3495915; doi:10.1371/journal.pone.0049292)
Supplement: Table S11 — Summary of primer sequences used in qRT-PCR analysis of selected genes. (DOCX) [file pone.0049292.s011.docx]

**Table S11. Summary of primer sequences used in qRT-PCR analysis of selected genes**

| **PaNo** | **Putative protein function** | **Primer** | **Sequence** |
| --- | --- | --- | --- |
| *Pa_1_16400* | PaCTR1, copper transporter | Pa_1_16400-1 | CTCACAATGTCACACTCC |
|  |  | Pa_1_16400-2 | TTAGCGATCAGTGCTACC |
| *Pa_2_4660* | PaSOD2, MnSOD | PaSod2-1 | TGGCTGCTGTTGAGAAGACC |
|  |  | PaSod2-2 | TCGGCCGTCTTCCAGTTGAT |
| *Pa_2_7310* | Putative fatty acid synthase alpha subunit, member of aflatoxin cluster | Pa_2_7310-1 | CACGGAAGCTCATGAAGTC |
|  |  | Pa_2_7310-2 | CAAGGATGATGCCAAGGTC |
| *Pa_2_7880* | PaMTH1, O-methyl transferase | Pa_2_7880-1 | CAACAAGGACGGCTATGCT |
|  |  | Pa_2_7880-2 | TCCTTCCACCGAATCTGAG |
| *Pa_2_9780* | PORIN | Porin-RT-for | TCTCCTCCGGCAGCCTTG |
|  |  | Porin-RT-rev | GAGGGTGTCGGCAAGTTC |
| *Pa_3_1710* | PaAOX1, alternative terminal oxidase | PaAox1-1 | CGCTGACAGAAGCTCAAT |
|  |  | PaAox1-2 | ATTCGTGGCGAGATCAAG |
| *Pa_3_10440* | PaCTR3 | PaCtr3-1 | GGCGATGTACTACAATGG |
|  |  | PaCtr3-2 | CCGTCGTCGTAATACTTC |
| *Pa_4_4770* | PaCTR2, copper transporter | Pa_4_4770-1 | CTGACGAGCACATACATGG |
|  |  | Pa_4_4770-2 | CGACGTTCATCGTCATCAC |
| *Pa_5_11970* | Putative ferric reductase transmembrane component | Pa_5_11970-1 | GACCAGTATGGCGAGGATG |
|  |  | Pa_5_11970-2 | CGTGGTATGTTCCGATGAG |

PaNo: accession number of the gene investigated in the *P. anserina* genome database. Putative protein function: short description or name of the gene investigated. Primer: names of the oligonucleotides used in qRT-PCR analyses. Sequence: primer sequences of used oligonucleotides.
